# An Update on the Use of Natural Pigments and Pigment Nanoparticle Adducts for Metal Detection Based on Colour Response

**DOI:** 10.3390/bios13050554

**Published:** 2023-05-18

**Authors:** Raspati D. Mulyaningsih, Rimadani Pratiwi, Aliya N. Hasanah

**Affiliations:** 1 Master Program in Pharmacy, Faculty of Pharmacy, Universitas Padjadjaran, Sumedang 45363, Indonesia; raspati17001@mail.unpad.ac.id; 2 Department of Pharmaceutical Analysis and Medicinal Chemistry, Faculty of Pharmacy, Universitas Padjadjaran, Sumedang 45363, Indonesia; rimadani.pratiwi@unpad.ac.id; 3 Drug Development Study Centre, Faculty of Pharmacy, Universitas Padjadjaran, Sumedang 45363, Indonesia

**Keywords:** natural pigments, metals, colourimetry

## Abstract

Natural pigments occur in plants as secondary metabolites and have been used as safe colourants in food. Studies have reported that their unstable colour intensity might be related to metal ion interaction, which leads to the formation of metal–pigment complexes. This underlines the need for further investigations on the use of natural pigments in metal detection using colorimetric methods, since metals are important elements and can be hazardous when present in large amounts. This review aimed to discuss the use of natural pigments (mainly betalains, anthocyanins, curcuminoids, carotenoids, and chlorophyll) as reagents for portable metal detection based on their limits of detection, to determine which pigment is best for certain metals. Colorimetric-related articles over the last decade were gathered, including those involving methodological modifications, sensor developments, and a general overview. When considering sensitivity and portability, the results revealed that betalains are best applied for copper, using a smartphone-assisted sensor; curcuminoids are best applied for lead, using a curcumin nanofiber; and anthocyanin is best applied for mercury, using anthocyanin hydrogel. This provides a new perspective on the use of colour instability for the detection of metals with modern sensor developments. In addition, a coloured sheet representing metal concentrations may be useful as a standard to support on-site detection with trials on masking agents to improve selectivity.

## 1. Introduction

Metals are elements that play important roles in the pharmaceutical industry. They are widely used in manufacturing as packaging material and equipment [1]. Some essential metals are critically important for physiological and biochemical functions of the human body, namely sodium (Na), potassium (K), magnesium (Mg), calcium (Ca), iron (Fe), manganese (Mn), cobalt (Co), copper (Cu), zinc (Zn), and molybdenum (Mo) [2,3,4,5]. Their deficiency or excess can lead to metabolism disturbances and cause various diseases [6]. Metals with atomic numbers greater than 20 and with an elemental density greater than 5 g/cm^3^ are called ‘heavy metals’, such as lead (Pb), cadmium (Cd) and mercury (Hg) [7]. Heavy metals are classified as dangerous elements due to their non-biodegradable properties, potential bioaccumulation, and toxicity when present in large amounts [8]. They may enter the human body through the ingestion of contaminated food and water, the inhalation of polluted air and through skin contact in agricultural, pharmaceutical, manufacturing, residential, and industrial areas [9]. Despite normally occurring in nature, metals may present as contaminants in canned food and fishery products, in agricultural crops from metal-concentrated soils, in tap-water, and in direct groundwater, especially around mining areas. Metals are mainly transported by water, are accumulated in the sediments of water bodies, and are highly concentrated in raw sewage [10,11,12]. Early detection and control in various materials of the environment become important as a measure of health protection.

Metals are conventionally quantified by instrumental analysis, such as atomic absorption spectroscopy (AAS) [13], flame or furnace spectroscopy [14], inductively coupled plasma mass spectrometry (ICP-MS) [15], energy dispersive X-ray fluorescence spectrometry [16], etc. These conventional techniques provide a highly selective and sensitive detection of trace metals, up to nanogram levels. Unfortunately, most of them cannot meet the needs of rapid detection since they lack portability and require a skilled workforce, time, and are relatively costly to operate [17]. The techniques were later modified to develop a field-portable analyser, offering a rapid, relatively inexpensive, and simple method compared to laboratory instruments [18]. Metals are known to be detectable through basic analysis principles, namely voltammetry [19], fluorescence-based sensing [20,21] and colourimetry [22]. Voltammetry-based metal detection can detect metals at the part per billion (ppb) level, but some electrodes lack specificity and reproducibility, and may form intermetallic compounds [23]. Fluorescence-based sensing is reported to be cost-effective by some research groups, but often undergoes a fluorescence quenching response that causes unsatisfactory selectivity for some analytes [17]. In recent years, developments in colorimetric portable analysers have been emerging. Colorimetric detection and sensor arrays may be an alternative to other methods, being lightweight and portable, requiring minimal instrumentation, responding to a wide range of analytes, and allowing a possible device fabrication with simple printing or integration with smartphone technology [24]. Changes in optical properties caused by metal reactions in colourimetry may result in colour changes that can be observed by the naked eye as a qualitative assessment and be detectable by visible light analysis [25,26]. Several materials have been developed in colorimetric detection, such as nanoparticles, carbon nanotubes, mesoporous materials, synthetic pores, conjugated polymers, and dyes [26]. However, some portable devices require reagents and materials that may be hazardous; hence, the proper disposal of waste must be considered [18].

Natural pigments are coloured compounds sourced from plants and are known to generate absorption when exposed to light in the ultraviolet and visible region [27]. Natural pigments are commonly used as food colourants, since their use is considered safe [28]. Some dyes made of natural pigments have shown a potential application in colorimetric metal detection. Studies have reported that metal ions can form complexes with dye molecules, such as betalains with nickel and copper [29], curcuminoids with lead [30], anthocyanins with iron [31], and carotenoids with copper, zinc, and lead [32]. These studies proved that natural pigments may also be alternative reagents for portable metal analysers. Unfortunately, there has not been a review that discusses the use of natural pigments in metal detection, the types of metals that can be detected, and which samples it can be applied to. This information underlies the further development of a pigment-based metal detector to detect metals at their lowest limits using a selective pigment. This paper aimed to discuss the use of natural pigments as reagents for portable metal detection based on published experimental studies and reported limits of detection (LOD) on various samples in order to determine which pigment is best for the detection of certain metals. We gathered recent updates on colorimetric metal-related articles over the last 10 years, including methodological modifications, sensor developments, and comparative results on real samples, with a general overview on natural pigment uses. Colour responses reported by all references in the articles may be mentioned as “colour changes” referring to significant change of colours, e.g., red to yellow; “colour stability/instability” referring to the ability to maintain its colour properties over time, tested from any kind of discolouration; “colour retention” referring to the ability to maintain the colour from fading; and “colour intensity” referring to the degree of brightness or dullness of a colour.

## 2. Natural Pigments for Metal Detection

**Figure 1:**
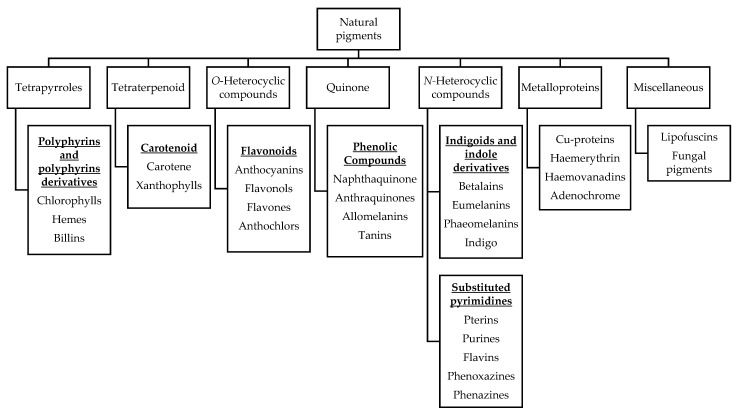
Natural pigments classified by structural affinities.

Natural pigments are secondary metabolites of plants that contribute to plant growth and development, photosynthesis, vegetation that attracts seed carriers and pollinators, and resisting biotic/abiotic stress [33]. Pigments are mainly distributed in fruits, flowers and leaves, and their main components are chlorophyll for green; carotenoids for yellow, orange, and red; flavones for yellow; anthocyanins for red, purple, and blue; and betalains for yellow and reddish-purple [34]. Based on structural affinities, naturally occurring pigments in biology are classified into six classes, namely tetrapyrroles, tetraterpenoid, *O*-heterocyclic compounds, quinone, *N*-heterocyclic compounds and metalloproteins, as shown in Figure 1, along with major examples of each class, as previously reported by Hendry [35].

**Figure 2:**
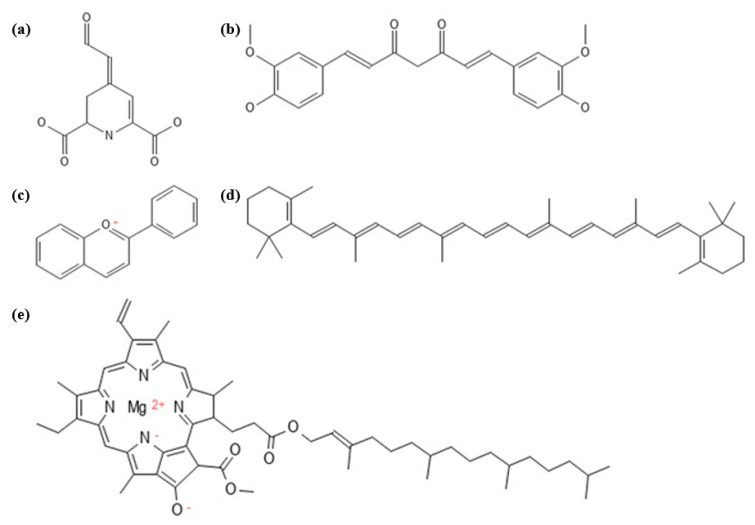
Generic chemical structures of natural pigments: (**a**) betalamic acid, the core structure of betalains; (**b**) curcumin, one of the components in curcuminoids; (**c**) flavium, the core structure of anthocyanin; (**d**) β-carotene, one of the components in carotenoids; and (**e**) chlorophyll.

In plants, widely known natural pigments are classified into three groups, namely tetrapyrroles, tetraterpenoids, and benzopyran derivatives, according to a diversity of structures and sources [36]. Tetrapyrroles are mostly known for chlorophyll, and tetraterpenoids for carotenoids, while benzopyran derivatives are known for anthocyanins and other flavonoids [37]. Another less-addressed pigment group, betalains, is limited and mainly contained in the order Caryophyllales [38]. The chemical structures of natural pigments are shown in Figure 2. These pigments are constituted by phenolic hydroxyl groups and are capable of exhibiting antioxidant activity. They are also able to chelate metal ions as pro-oxidants that are involved in the production of free radicals [37,39,40]. Pigment–metal interactions were first reported in 1919, when it was noted that flavonoles and flavonones in flowers are originally colourless but form a coloured compound (co-pigmentation) as a result of complexation with metal ions [41]. Anthocyanin was found to form a blue colour after being exposed to magnesium; thus, the metal–complex theory was first proposed [42,43]. Colour stability studies against metal exposure were also performed to observe colour changes among the pigments. The colour stability of the betalain pigment from Cactacea fruit was found to decrease after exposure to iron and chromium [44]. Metal ions were found to accelerate the oxidation and colour loss of betalains [45]. Similar results were also obtained in another study that mentioned the accelerated degradation of anthocyanin in red cabbage by metal ions (Al^2+^, Ca^2+^, Fe^2+^, Sn^2+^) [46]. This result corresponded with a previous experiment where the colour stability of anthocyanin in morning glory was greatly affected by Fe and Zn [47]. On the other hand, the presence of metal ions (Fe^2+^, Fe^3+^, Cu^2+^, Al^2+^, Al^3+^) in anthocyanin-extract mixtures has been patented as means of stabilising the colour by forming anthocyanidin complexes for food application [48]. The colour changes and stability studies showed that the pigments react to certain metal ions. This phenomenon underlies further investigations, as certain amounts of metal may cause colour changes, and the specific colour changes of pigments may be a sign of the presence of metal ions. Hence, colorimetric metal detection using colour-changing pigments were investigated.

Colorimetric detection is a solution-based assay to determine the concentration of coloured compounds by assessing the change of absorbance or reflectance of an analyte [49,50]. The determination is conducted using the absorption of the coloured compound that can be detected by the naked eye, within the visible wavelength (400–800 nm) or identified and quantified by instrumentation [37]. The instruments detect colours on specific wavelengths that generate maximum absorptions. The chromophores or functional groups of some structural features are responsible for giving colour to a compound, which can be detected and quantified at a specific wavelength. The analysis is conducted using a spectrophotometer for instrumental detection and quantification [51], which may be paired with various detectors, depending on the wavelength range; for colorimetric detection, ultraviolet-visible (UV-Vis) absorption, emission, fluorescent and infra-red spectrophotometry is mostly used [52]. On the other hand, modified forms of non-instrumental colorimetric detection have also been applied in recent studies, such as a regular paper-based analytical device [53] and a microfluid paper-based analytical device [54]. During sample application, these devices do not require electromagnetic instrumentation to detect a colour change and predict concentrations. Instead, the determination relies on observing the colour change using the naked eye and comparing the results to a coloured sheet standard [55]. As mentioned above, the colour-changing reaction between natural pigments and certain metal ions gives a new perspective on the use of natural pigments as a reagent in colorimetric detection, especially in non-instrumental devices.

**Figure 3:**
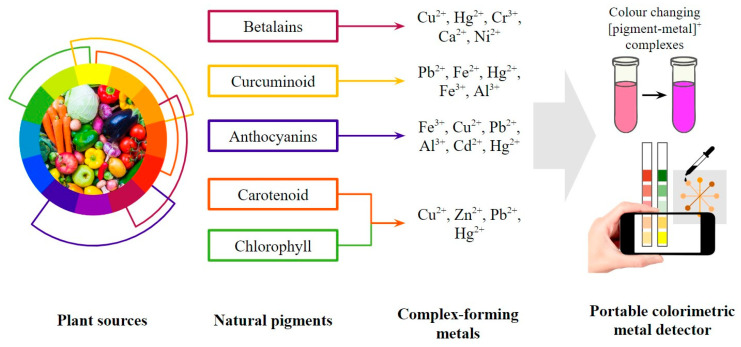
General scheme of portable colorimetric metal detector using natural pigments.

The utilization of natural pigments also gives hope to green material-based colorimetric probes as an alternative to the complex material preparation processes and the potential hazards of synthetic dyes. Natural pigments are less allergenic, less toxic, and generally more compatible with the environment than synthetic ones [56]. Along with a large number of industrial pollutants, some synthetic pigments also promote toxicity, mutagenicity, and carcinogenicity. The higher environmental compatibility of natural pigments makes them more sustainable with minimum damages and health risks during applications [57]. Over the last decade, 18 studies have reported updates on natural pigments and the development of sensors in various forms. Some pigment classes were selected in recent studies on colorimetric detection, such as betalains in solution-based form [58], curcuminoids in membrane form [59], anthocyanin in dipstick form [60], etc. The selection was based on the potential to form coloured compounds with bivalent and trivalent metal ions and the selectivity of each pigment. This review will mainly focus on betalains, curcuminoids, anthocyanins, carotenoids, and chlorophyll, as previously reported in these studies and mapped out in Figure 3.

## 3. Betalains

Betalains are pigments considered as markers of the order Caryophyllales and are abundant in *Beta vulgaris* L. [61]. The betalains isolated from prickly pear (*Opuntia ficus-indica* (L.) Mill.) were identified as indicaxanthin and betanin [62], while those in *Amaranthus caudatus* L. were identified as amaranthine, isoamaranthine, betanin and isobetanin [63]. According to their colour range, betalains can be divided into two groups: betacyanins for red-violet and betaxanthins for yellow-orange [64,65]. All compounds in betalain group contain a core structure of betalamic acid [4-(2-oxoethylidene)-1,2,3,4-tetrahydropyridine-2,6-dicarboxylic acid] (see Figure 2). The formation of betacyanins, which are glucosyl derivatives of betalains, is initiated by condensation with cyclo-dioxyphenylalanine (cyclo-DOPA), while betaxanthins are formed by condensation with amino acids [66]. Betalains are commonly used as food colourants for their red-violet and orange colours [67]. Any possible colour change of the colourant is anticipated by preliminary stability studies, since metal-based primary packaging is used with various coloured products. Studies have been conducted to investigate the effects of physicochemical influences on the pigments, including exposure to metal ions. Stability studies of the pigment can provide information on any colour change caused by metals, starting from a trace amount, and support the idea of using betalains for metal detection. It was found that metal ions caused colour instability in betalains (Table 1). Absorbance and wavelength shifts were observed with a UV-Vis spectrophotometer in order to detect the slightest colour change after metal exposure.

In 1977, Kuusi et al. reported that a significant loss of colour intensity in red beet juice was essentially dependent on pH and mainly occurred at pH 3.4 with the addition of Cr^3+^, compared to Fe^2+^, Sn^2+^ and Al^3+^ [68]. The metals did not cause great changes in hue; instead, high precipitation was obtained in Fe^2+^ samples under acidic conditions [68]. The presence of metal ions (Cu^2+^, Fe^3+^) was also reported to decrease the degree of colour regeneration of betanin pigment in red beet juice [69], but precipitation was not reported. Precipitation by Fe^3+^ occurred at pH 3 and was related to the strong degradation of betanin [29]. These different results may be linked to the different pH conditions in these studies, since the first study was performed at pH 4.0 and 6.0 [69]. Remarkable changes in the UV-Vis spectra of betanin solution were also obtained after exposure to Cu^+^, Cu^2+^, Hg^2+^ and Ni^2+^, suggesting the formation of a pigment–metal complex that led to either a bathochromic or hypochromic effect [29,69,72]. A stability study on 2-decarboxy-betanin in aqueous-organic solution confirmed that Cu^2+^ induces pigment degradation, indicated by a new absorbance band in the visible spectra, in the range of 390–500 nm [70]. The band was possibly generated by betacyanin derivatives, as recent studies have reported that betacyanin oxidation leads to the production of yellow-coloured decarboxylated and dehydrogenated betacyanins [29]. Under weak alkaline conditions (pH 7–8), Skopińska et al. [70] observed a large maximum absorption shift, which corresponded with the observation by Wybraniec et al. [29] that the fastest spectral changes of red beet in Cu^2+^ occurred at a pH of 8, with 0% colour retention level, followed with a pH of 3 at 25%. In either a pH of 3 or 8, an increase of methanol and ethanol was also found to contribute to betanin degradation [70]. The metal-induced and pH-dependent instability of betalains shows that they respond to metal exposure, resulting in chemical degradation and spectral shifts at certain pH levels. The formation of a pigment–metal complex possibly underlies the colour degrading effect of betalains, supporting the idea that the colour-changing effect of metals against the pigment is possible for colorimetric metal detection. However, further studies are required to determine whether observation by the naked eye are possible and at which concentration level a colour change occurs, as well as developing a portable detector.

Recent studies have reported that different types of metal detectors have been developed using red beet pigment (Table 1), namely a functionalised nanoparticles [71] and a smartphone-based analyser [58]. Formerly in 2013, Gonçalves et al. used betanin (additive E-162) pigment as a colorimetric sensor for the detection of calcium dipicolinate (CaDPA) in bacterial spores [73]. The pigment was claimed to be responsible for the red-magenta colour of red beetroot. They developed an aqueous-based method that formed a betanin–europium(III) complex ([Eu(Bn)]^+^), changing the colour from magenta (betanin) to orange ([Eu(Bn)]^+^). An amount of CaDPA was then added to the complex solution, resulting in a magenta colour. Gonçalves et al. claimed that CaDPA competes with betanin for Eu^3+^ ions, releasing the betanin due to the formation of a [Eu(DPA)_n_]^3−2n^ complex. This was followed by a reversed colour change from orange to magenta as a sign that betanin molecules were released from the complex [73]. The study did not report on whether betanin formed a complex with Ca^2+^ or not, as the structure of the complex was estimated by Raman spectroscopy associated with theoretical calculation. However, it is noteworthy that the pigment–metal complex of [Eu(Bn)]^+^ was successfully formed using a molar-ratio estimation (1:1) with a stability constant of 1.4 × 10^5^ L mol^–1^. The colour intensity of the complex solution was quantitatively determined by UV-Vis spectrophotometry, and the colour change was observable by the naked eye [73].

In the following year, Barman et al. developed a colorimetric assay specifically purposed for mercury (Hg^2+^) detection using synthesised betacyanin-gold and betacyanin-silver nanoparticles [71]. Betacyanin was formerly extracted from *Beta vulgaris* through aqueous thermal extraction and filtration. The extract was used to synthesise gold/silver nanoparticles that were intended to chelate Hg^2+^ in water samples by a capping mechanism. Visible colour changes were obtained after the addition of Hg^2+^: red to violet for betacyanin-Au and yellow to purple for betacyanin-Ag. In this mechanism, betacyanin acted as a chelating ligand to Hg^2+^ and facilitated the aggregation of nanoparticles. The maximum absorption of both peaks in the UV-Vis spectra shifted to a higher wavelength, followed by a decreasing absorption coefficient. Both colour changes were also observable by the naked eye. Barman et al. used a fluorescence sensor to sense Hg^2+^ in aqueous solution, as the betacyanin–gold nanoparticles were fluorescent and their fluorescence decreased significantly when the metal was added. The strong metalophilic interaction of Hg-Au and Hg^2+^-carboacylate induced luminescence quenching in the spectrofluorometer. The sensor was highly sensitive and generated fluorescence intensity for quantitative Hg^2+^ determination, resulting in a minimum detection of Hg^2+^ at 25 µM and a maximum detection at 50 µM [71]. The instrumental uses in this development made it less practical for rapid and portable detection, since UV-Vis spectrophotometers and spectrofluorometers are only used in laboratories.

Later in 2019, Cao et al. constructed a Cu^2+^-selective colorimetric sensor for water samples using a red pigment that was originally extracted and powdered from red beet [58]. Under alkaline conditions, the redox reaction and chelation of Cu^2+^ by the red beet pigment was expected to form a colour change within 10 min of incubation, from purple to orange-red solution at pH 9. The sensor was built into a smartphone-based analyser with a developed application for androids that allowed detection by taking pictures of the sample using the phone’s camera and selecting the area for analysis. The application generated a quantitative result based on the visual colour change of the solution, which linearly interpreted the colour intensity into metal concentration. This method allowed the detection of at least 0.84 µM of Cu^2+^ ions in water samples. According to the reported visual observations, the colour change from purple to orange red slightly changed as the metal concentration increased so that, for naked eye observations, the orange-red colour could be seen above 20 µM.

The sensors used in the two methods above are UV-Vis spectrophotometer, spectrofluorometer for fluorescence-based sensing, and a smartphone-based analyser using a phone camera. In terms of portability, the smartphone-based method is the most portable compared to the other methods. The method used by Barman et al. may possibly be applied to portable techniques, but it requires further development to allow for rapid and portable detection. The smartphone analyser allows for portable use in the rapid detection of metals in solution samples, as Cao et al. also reported recovery levels of 100–109% [58]. It is important to note that different environmental lighting conditions, phone types, and camera resolutions may result in different quantitation since the sensor is based on the pictures taken. Furthermore, accuracy, precision and robustness studies are required for different devices. Compared to other instruments, colour intensity can be accurately measured by UV-Vis spectrophotometry as it relies on absorbance being interpreted into coloured compound concentration levels at specific wavelengths. UV-Vis does not support portable analysis but is used in comparative studies for colorimetric method development. Spectrofluorometry is highly beneficial for fluorescent compounds, but not all pigment–metal complexes are fluorescent. From the studies discussed above, it has been proven that betalain–metal complexes are coloured and that observation by the naked eye is possible, although the intensity and colour changes are dependent on the metal.

## 4. Curcuminoids

Curcuminoids are a group of secondary metabolites that have been attributed to the various pharmacological activities of turmeric, which consists of curcumin and two related compounds, demethoxycurcumin and bidemethoxycurcumin [74]. Curcumin [1,7-bis(4-hydroxy-3methoxyphenyl)-1,6-heptadiene-3,5-dione] (see Figure 2) is the main constituent of the turmeric rhizome (*Curcuma longa* L.) and has been used as a food additive for colour and as a spice [75]. The curcumin content varies from 0.6–5% of the *Curcuma longa* rhizome (dry mass) [76], 40% in turmeric oleoresin [77], and 0.74–1.23% in *Curcuma xanthorriza* [78]. The application of curcumin in colourimetry was first investigated as a strip test, where the colour changed from yellow to reddish brown [79,80]. The ability to form a complex of curcumin was also investigated based on its theoretical ability to bind to metal ions because of the acetylacetonate (acac-) ligand contained in curcumin, and thermodynamically, the complexes may be stable in polar media [81]. Krishnankutty et al. confirmed that curcuminoids can bind to Ni^2+^, Co^2+^, Zn^2+^ and Pd^2+^ as the ions structurally replaced the enol proton, with the formation of a stable six-bonded ring involving metal chelation [82]. Curcumin was also studied as a potential Fe^3+^ chelating agent by the formation of iron-curcumin [FeH_2_CU(OH)_2_] and iron-diacetylcurcumin [FeDCU(OH_2_)] complexes [83]. Curcumin complexes were found to be stable at a 2:1 (ligand:metal) molar ratio with Ga^3+^, forming a [Ga(Curcumin)_2_]^+^ complex [84,85].. The stoichiometric ratio indicates that a certain amount of the metal can be fully bound to a certain amount of curcumin, supporting the idea that the determination of the metal concentration in a matrix is possible using curcumin.

Recent studies have reported that curcumin–metal complexes are colour-changing, resulting in the further investigation of curcumin in colorimetric analysis. In 2014, solution-based colourimetry using curcumin-stabilised gold-nanoparticles for mercury (Hg^2+^) detection showed positive results, marked by a colour change from reddish wine to a light blue solution at pH 7.4 and further analysed using UV-Vis spectrophotometry [86]. Compared to the betacyanin gold-nanoparticles in the study by Barman et al. [71], the LOD using curcumin is much lower than betacyanin, as it allows for the detection of mercury at 2 µM [86], while betacyanin allows for a minimum detection at 25 µM. The selectivity of curcumin makes it tricky to avoid false positive results for metal detection in alkaline solutions, since it can naturally change colour at pH values above 7.4 and form metal hydroxide precipitates [59], although the stabilizing method by Kumar et al. claims that the nanoparticles allow for improved physical stability at pH 7.4 based on UV-Vis spectrophotometry analysis where the band was centred at 523 nm [86]. Unlike the naturally occurring curcuminoids, pure curcumin is highly prone to chemical degradation in alkaline solutions and tends to crystallise in acidic solutions [87]. Metal detection was then developed using synthesised curcumin-based materials, such as nanofibers, nanoparticle films, cryogels, biofilms, and zein membranes, as summarised in Table 2. Fabricated modification is one of the alternatives to improve the bioavailability, solubility, and stability of curcumin; it also tends to improve the chelating ability, sensitivity, and selectivity of curcumin-based sensors [88]. In terms of portability, nanofiber-type materials are highly portable as they are ready to use in fabricated form, easy to handle, simple and stable, and suitable for rapid colorimetric detection with visual observation [89]. For the detection of lead ions, three sensors were developed with different results: curcumin silver-nanoparticles (AgNPs-CUR) for solution-based sensors, and cellulose nanofibers and starch cryogel for fabricated sensors (Table 2). The curcumins used in the studies were extracted from fresh turmeric (*Curcuma longa* L.) [90] and powdered turmeric [30,91,92]. Curcumin contains a 1,3-diketone group including a stable ketoenol tautomeric state that allows it to form a strong complexation through a chelating mechanism with Pb^2+^ ions. Positive results were observed for the presence of Pb^2+^ using AgNPs-CUR, marked by a colour change from yellow to orange at a pH of 6, followed by floccular precipitates within 20 min [90]. Curcumin cellulose-nanofibers also changed colour from yellow to orange red at a pH of 5 [30,91]. However, in a study by Phatthanawiwa et al., a curcumin–starch cryogel did not change colour in the presence of Pb^2+^ ions at a pH of 2 [92]. In a similar experiment at a pH below 4, there was also no remarkable visible colour change in the nanofibers [30]. These results confirmed that the colour-changing property of curcumin is dependent on pH for Pb^2+^ detection, as an orange-red colour is observable at pH values above 5. A red-brown colour was also observed for Fe^3+^ at pH 2 [92], although it was claimed to be selective against Ni, Cu, K, Mn, Cr, Mg, Zn, Na, Ca, Ba, Cd and Co ions, and was found to be the most sensitive for Fe^3+^ [30,59,91,92]. Fe^2+^ detection was also performed using a biofilm composed of mixed curcumin, banana (*Musa acuminata x balbisiana*) and aloe vera (*Aloe barbadensis*); a greenish-brown colour was observed and confirmed by UV-Vis spectroscopy, with a wavelength shift from 426 nm to 410 nm [88].

The main challenge of using curcumin as a colorimetric indicator for metal detection is its naturally pH-dependent property, which may lead to false positives, especially in alkaline conditions, although some studies reported that the methods are selective for certain metals. Based on the reports above, a fabricated form is preferable than a solution-based form as it improves curcumin stability. All colour changes in the fabricated forms are observable by the naked eye, and the colour intensity is positively linear to the metal concentration [92]. In terms of sensor stability, cryogel strips were reported that the size became smaller and harder under ambient conditions but was biodegradable, as almost 100% weight was lost within 15 days [92]. In contrast, the curcumin-mixed biofilm showed a lower biodegradation rate than the cryogel, at 1.28% in one week [88].

## 5. Anthocyanin

Anthocyanin is a glycosylated anthocyanidin that naturally forms as a red pigment contained in red cabbage (*Brassica oleracea* var. capitata f. rubra), red raspberry (*Rubus idaeus*), eggplant (*Solanum melongena* L.) [93], roselle flowers (*Hibiscus sabdariffa*) [94] and hydrangea flowers (*Hydrangea macrophylla*) [95]. Anthocyanidin is the aglycone of anthocyanin [96]. Glycoside derivatives of nonmethylated anthocyanidin are the most common in nature and found in 80% of pigmented leaves, 69% of fruits and 50% of flowers [97]. Anthocyanin is commercially available as a purple food additive (E163) derived from grape skin. It is used as a colourant in coloured jam, confectionaries, and beverages. It is a bioactive pharmaceutical compound, known as a nutraceutical, with potential antioxidant, anticancer, antiobesity, and neuroprotective benefits, as well as promoting cardiovascular health and visual health [96]. Anthocyanidin belongs to the flavonoid group, a larger polyphenol group capable of absorbing light when exposed to the UV and visible regions [98]. The colour exhibited by anthocyanin depends on the conjugated bonds that result in different coloured pigments, mainly red, blue, and purple [96]. The co-pigmentation of anthocyanins helps to stabilise the colour by forming noncovalent complexes with metallic ions, organic acids, and flavonoids [99] or subsequent changes of anthocyanin optical properties [100]. It occurs naturally in flowers [43], vegetables and fruits [101]. Anthocyanins can also act as a co-pigment in self-association co-pigmentation if both parties are present in AH^+^, A or A^−^ form [96]. Co-pigmentation in general can intensify the colour and cause spectral shifts in spectrophotometry [96,102]. For example, it forms a stable and intense blue ferric anthocyanin pigment at a molar ratio of 2:1 (ligand:metal ion) and a pH of 4–5 [99]. Anthocyanidin consists of an *o*-dihydroxyl group bonded to a B-ring structure, which allows it to serve as a ligand and form a coloured complex with metal ions [103,104]. The formation of anthocyanin–metal complexes is mainly affected by the oxidation state of the metal ions [105]. However, co-pigmentation with metal ions also negatively affects the stability of anthocyanins, as reported for Sn^2+^ and Al^3+^, inducing significant colour loss [46]. Stability studies have shown that metal exposure causes a colour change, either degradation/loss or intensity improvement. This phenomenon supports the idea of metal analysis using colorimetric detection in that anthocyanin exhibits a colour change whenever it is exposed to metal ions. In addition, it occurs under pH influences that make it more specific for certain metals.

Experimental studies have reported the development of colorimetric detection using anthocyanin, as summarised in Table 3. Khaodee et al. reported a solution-based method using cyanidin extract from red cabbage [106]. Cyanidin is an anthocyanin derivative that naturally presents in red violet or purple. Positive results in this study were marked by a colour change from violet to blue. Fe^3+^, Cu^2+^, Pb^2+^ and Al^3+^ were optimally detected at pH values of 4, 5, 6 and 7, respectively, followed by quantification using UV-Vis spectrophotometry. The detection limit of each metal was at the micromolar level and results were observable by the naked eye. The colour change of all metals tested were also similar, although the method involved potassium fluoride and dimethylglyoxime as masking agents to mask unexpected interfering ions [106]. Cyanidin was investigated further in the colorimetric analysis of Fe^3+^ ions in a fabricated form. Khattab et al. developed a dipstick sensor, where a phenolic cyanidin chromophore was imprinted onto nanoparticle strips for on-site detection in water samples [60]. Ferric ions were detected in the range of 179–7162 µM and the colour change could also be observed by the naked eye. The study also reported that ferric detection was best performed at pH values of 4–6, with the highest absorbance intensity observed at pH 5. However, the colour changes between these two studies were not identical, although both pigments were extracted from red cabbages. Khaodee et al. observed pink to violet to blue changes in hue, while Khattab et al. observed a white to pink colour change. The colour changes of anthocyanin in aqueous media were formerly reported to be pH dependent due to a variety of molecular transformations. It is red in a strong acidic medium, as the flavylium cation; pink in weak acid; purple/violet in neutral media, as the anhydrobase; blue-green in weak alkaline, as the anhydrobase anion; and yellow in strong alkaline, as chalcone [107,108]. Anthocyanin may also be present as the colourless carbonyl pseudo-base in acidic solutions (pH 4–5) [108]. Cyanidin naturally presents a red-violet colour in acid and changes to purple blue as the pH increases to alkaline [109]. The metal chelation of Fe^3+^ by cyanidin caused a bluish colour as a consequence of the interaction between quinoidal and the anhydrobase ion [109], followed by the bathochromic and hyperchromic effects of metal acylation [93]. Thus, the different results were possibly caused by the pH-dependent anthocyanin structural form, since chelation of Fe^3+^ is dependent on the position of the active sites of oxygen atoms and the cations of the structures [108].

Eco-friendly fabricated hydrogel strips were also developed for Cd and Hg detection in water samples. The strips changed colour from white to green and blue to indicate a positive result for Cd^2+^ and Hg^2+^, respectively [110]. Fabricated forms are more portable than solution-based forms and support naked eye observations for qualitative purposes. The challenge of this type of observation is that it requires a colour standard for comparative interpretation to determine the metal concentration without laboratory instruments for quantitative purposes. In the anthocyanin studies mentioned above, quantitative analysis was performed using UV-Vis spectrophotometry, which is non-portable for on-site analysis. Another colorimetric method was developed to allow quantitative analysis. A smartphone-assisted colorimetric determination of Fe^3+^ in aqueous media was developed using an anthocyanin extract from *Ruellia tuberosa* L. as a green indicator [31]. It formed a red anthocyanin–metal complex within 40 min of incubation at a pH of 1. The LOD for Fe^3+^ in this method was also lower than in the previous methods, with 0.54 µM reported by linear-graph estimation and noticeable visual observation at 35 µM. This was due to the highly acid condition, which stabilised the red anthocyanin. The metal concentration in the samples was determined by capturing colour changes with a smartphone and analysing the intensity with ImageJ using the RGB system. The colour intensity was interpreted as a concentration level using a calibration curve in Excel [31]. This study eliminated interfering environmental lighting using a control lightbox where the pictures were taken. Digital evaluation with the ImageJ RGB system can be used as an alternative to the spectrophotometer since it uses affordable imaging devices and does not rely on signals. Users can perform colorimetric analysis anywhere using the software installed in the computer. Unfortunately, the software interpretation relies on the shape uniformity and colour homogeneity of the sample and may be unsuitable when analysing objects with irregular colour distributions and shapes [111].

## 6. Carotenoids and Chlorophyll

Other natural pigments, namely carotenoids and chlorophyll, have previously been reported as potential metal detectors for Cu, Zn, Pb and Hg, as shown in Table 4. The carotenoids are a class of tetraterpenoid pigment group widely used in the food industry. β-carotene is the most abundant carotenoid in food with the highest activity of provitamin A, such as carrot, tomato, and pumpkin [112]. Carotenoid from wild carrot (*Daucus carota*) has reportedly been used for the detection of heavy metals [32]. The study used a cultured cell-bound carotenoid for the detection of Cu^2+^, Zn^2+^ and Pb^2+^ ions, allowing for a minimum detection of 0.157 µM, 0.153 µM and 0.048 µM, respectively. Exposure to heavy metals caused significant absorbance changes, with an optimum response time of 40 min. Significant shifts in spectrophotometry mark a potential colorimetric-sensor property, but the mechanism of reaction behind it is not yet fully understood. It has previously been reported that the chemical stability of carotenoids is affected by metal ions, mainly Cu^2+^, Fe^3+^ and reactive oxidative oxygen species, which accelerate the metabolism of the carotenoid in plants, reducing its bioavailability [113,114].

Chlorophyll-based silver nanoparticles were synthesised and investigated to create another colorimetric sensor for the detection of mercury. Chlorophyll-based silver nanoparticles were found to be highly dispersed due to the electrostatic repulsion of the negatively charged methyl (-CH_3_) and methylene (-CH_2_) groups on the surface of the nanoparticles [115]. The Hg^2+^ ions initially bind to the methyl groups on the surface of nanoparticles and form a methylmercury ([CH_3_Hg]^+^) which is highly oxidative [116]. The oxidation of metallic silver to silver ion occurs with the reaction of Hg^2+^ in the methylmercury, thus the colour of the nanoparticles changes from brown to colourless [117]. The nanoparticles resulted in a minimum detection of 2.7 µM and 60 µM when observed by UV-Vis spectrophotometry and the naked eye, respectively. Within five minutes of incubation, the colour of the chlorophyll nanoparticles gradually changed from brown to light brown or colourless with the increases in mercury concentrations [117]. Compared to other Hg^2+^ pigment sensors, this method has a higher LOD, which is less favoured for the detection of the smallest amounts of metals. However, this method is still a good option since it is selective against Na, K, Ba, Zn, Mg and Ca, and the pigment was extracted from mint leaves that are available in large quantities.

**Table 4:** Studies on metal ion detection by carotenoids and chlorophyll.

Pigment	Sample		Metal Selectivity	Colour Change	Stability	Limit of Detection	References
	Real	Synthetic					
Cell-bound carotenoid extracted from *Daucus carota*	Not tested	Cu/Zn/Pb solution added into media containing cell suspension	Cu^2+^	Increased absorbance	Maximum optical density at 40 min of exposure	0.157 µM (instrument measurement)	[32]
			Zn^2+^	Increased absorbance	Maximum optical density at 40 min of exposure	0.153 µM (instrument measurement)	
			Pb^2+^	Increased absorbance	Maximum optical density at 40 min of exposure	0.048 µM (instrument measurement)	
Chlorophyll-based silver nanoparticle (mint leaf extracts)	River water with pre-treatment of filtration	Hg^2+^ solution in ultrapure water	Hg^2+^	Brown to light brown or colourless	Not tested	2.7 µM (spectrophotometer UV-Vis), 60 µM (visual)	[117]

## 7. Comparative Result of Metal Detector Using Natural Pigments

The recent experimental studies summarised in Table 1, Table 2, Table 3 and Table 4 have shown that natural pigments can form metal–pigment complexes for metal detection. The colour intensity changes due to the formed complexes are marked by absorbance changes or visually fading colours. The metals detectable by natural pigments are Cr^3+^, Cu^2+^, Hg^2+^, Ca^2+^, Pb^2+^, Fe^2+^, Fe^3+^, Al^3+^, Cd^2+^ and Zn^2+^, with various colour changes. Most samples used in the studies were drinking water, tap water and pond water, since metallic pollutants mainly accumulate in water. Copper ions (Cu^2+^) were detectable in drinking water at the lowest concentration of 0.84 µM using a smartphone analyser by linear-graph estimation [58]. The developed method complies with the requirements for the detection of copper ions, as the WHO set the maximum copper contamination in drinking water as not more than 2000 µg/L (31 µM) [118]. In tap-water and pond-water, the lowest lead detection was obtained using anthocyanin with a minimum detection of 50 µM [106]. This showed that the smartphone analyser is highly sensitive and it is also applicable for tap-water and pond-water samples. However, each sensor has various advantages and disadvantages in terms of portability.

For lead-ion (Pb^2+^) detection in water, the lowest lead detection was obtained by curcumin-AgNP nanoparticles with a minimum detection of 13.6 µM [90], followed by anthocyanin extract with a LOD of 80 µM [106]. These results do not meet the requirements of WHO which state that the maximum lead contamination in drinking water is 10 µg/L (0.048 µM) [118]. However, the method can be useful for lead detection in irrigation water as a non-potable water source, where the maximum acceptable level is set at 5000 µg/L (24.1 µM) [119]. It can be used as an alternative method to evaluate the suitability of irrigation water for beneficial uses. A more recent study in 2022 showed that a lower detection limit was obtained using curcumin nanofibers at 9 µM visually and at 0.9 µM by spectrophotometer during the experiment [91]. The study was performed on rice samples with pre-treatments using aqueous solutions. According to the Codex standard limit, the maximum contamination of Pb in rice is 200 µg/kg [120]. This is lower than the LOD of curcumin nanofibers (18,600 µg/kg visually), indicating that the method requires sensitivity improvement. It is noteworthy that the main challenge of developing curcumin-based sensors is related to the physicochemical properties of curcumin. It naturally presents in vibrant yellow, is highly affected by pH, and the colour change after exposure to lead ions in low concentrations is not significant. Thus, visual observation can be difficult. A lower detection limit of 0.048 µM was formerly obtained using carotenoid by instrument measurement in 2014 [32], which may cover the requirements but the application in water using carotenoid has not been conducted; hence, further investigation is required. Curcumin nanofibers had the highest point for portability and lowest visual LOD, although the sensor stability has not been investigated in detail. It was only reported that the strip had fibrous morphology and became thicker after the addition of Pb^2+^ [91]. The strip stability as a sensor may require further studies, including storage monitoring in different environments, degradability, and colour stability after several washings. The selectivity of curcumin for lead detection against other metals may be challenging. As mentioned in Table 2, the colour changes mostly range around yellow to brown, orange, or red. However, it has been confirmed that the strip method is selective against other divalent cations: Cd^2+^, Ni^2+^, Ba^2+^, Mg^2+^, Zn^2+^and Ca^2+^ [91].

The lowest concentration of mercury ions (Hg^2+^) in water samples was detectable in the most recent study using anthocyanin hydrogel (LOD 0.2 µM by linear-graph estimation) [110], followed by curcumin–gold nanoparticles, with a visual LOD of 2 µM [86], compared to betalain (LOD 25 µM) and chlorophyll (LOD 2.7 µM with UV-Vis spectrophotometry, 60 µM visually) [71,117]. Mohan and Prakash also reported that a colour change at 10 µM can be visually observable, but the lowest concentration for naked eye observation was not reported [110]. For inorganic mercury, the maximum contamination in drinking water of not more than 6 µg/L (0.029 µM) is set by WHO [118]. The developed methods for mercury detection have not accomplished the requirements for detection in drinking water, as each LOD is lower than the maximum contamination limit. The challenge in developing colorimetric detection is that lower concentrations of Hg may not show significant changes in hue, thus leading to undetectable results. Sensitivity improvements may be the main parameter to assess in further developments. In terms of portability, anthocyanin hydrogel allows for portable use, except for naked eye observations that require a UV chamber. As an alternative, curcumin–gold nanoparticles provide naked eye observations without the need for additional instrumentation.

Fe^3+^ is detectable by curcumin and anthocyanin, but Fe^2+^ is only detectable by curcumin. The lowest detectable concentration of Fe^3+^ was obtained by anthocyanin using a smartphone-assisted sensor, with a LOD of 0.54 µM by linear-graph estimation [31], followed by a curcumin-loaded membrane at 7.16 µM by visual observation [59]. Iron (Fe) contained in natural freshwater ranges from 0.5–50 mg/L and is stated to be an essential element in human nutrition; thus, no guideline value was proposed by the WHO for iron in drinking water. However, The Joint FAO/WHO Expert Committee on Food Additives (JECFA) set a provisional maximum tolerable daily intake (PTMDI) of iron at 0.8 mg/kg body weight, and a 10% allocation of the PTMDI for drinking water gives a value of 2 mg/L (35 µM) [118]. Smartphone-assisted sensors and pigment-loaded membranes are both highly portable for rapid detection, although each method has different pros and cons. Curcumin-loaded membranes were also successfully developed to detect iron in samples, although the colour-change property can be tricky and less selective.

From all the methods mentioned above, there are two portable sensor developments that involve a smartphone. The first one was developed using betalain that supports direct quantitative analysis, and the second one was developed using anthocyanin which still requires an additional device. Compared to fabricated forms, smartphone analysers can last longer and are less likely to be degraded over time. In the study, Cao et al. reported that the sensor was constructed by taking images of a set of pigment solutions containing a series of copper concentrations. The phone was fixed on a holder at a 30 cm distance in front of the reaction system. The blue values in the RGB colour model from the images were obtained using Adobe Photoshop software. Later, the average blue values of every twenty points of a sample were fitted to a linear curve versus the metal concentration. According to the curve, a smartphone application based on an android system was developed and the programming language was JAVA [58]. This method will allow users to only install the application and enables them to perform tests on-site followed by quantitative estimation. We noticed that some interferences may affect the test results. A control lightbox is extremely important to block interfering lighting and the pictures taken are dependent on the camera resolution. This indicates that a minimum camera resolution and robustness between smartphones are necessary. Apart from the previous method, another smartphone-assisted sensor was also developed by Meelapsom et al. for iron detection including method testing on three different smartphones [31]. The role of the smartphone was to monitor the colour change of a pigment under the control lightbox. The images were interpreted using the RGB system in the ImageJ software in a separate computer. According to the sensor performances, the utilisation of ImageJ software allows for the stable interpretation of colour intensity [31]. It uses the RGB system to determine the shades in hues with the help of the lightbox that removes lighting interferences. Unfortunately, using Excel to determine the concentration can take some time, making it less rapid. Another alternative way to minimize lighting interference is by developing the software that allows for a calibration step. Image processing can be articulated into a few steps. It is initiated by the algorithm that filters out the background noise from the image, extracts the region of interest, followed by functions that cut out the background resulting in a high-contrast image with a black background [121]. Further studies should be performed in order to determine whether a calibration system can eliminate a control lightbox.

The possibility of detecting multiple metals using one type of pigment indicates that false positives/negatives may occur. In view of the fact that, in water samples, there could be multiple metals present and one ligand could potentially form a complex with many ions. In addition, colour changes were also found to be affected by pH, meaning that at certain acid or alkaline levels without metal exposure, a colour change may be noticed. Buffer selection can be tricky for pH-induced colour changes. For example, curcumin turns red in alkaline solutions. It potentially shows false positives for metals that change the colour of curcumin from yellow to red. Thus, an acidic condition is preferred. In selecting buffer solutions, compatibility of acid and metals should be considered. Phosphate, carbonate, and chloride anions form precipitates with Pb^2+^, while an acetate buffer can be an alternative [91]. False results can be anticipated by performing tests on a solution containing various metals under all pH levels to evaluate the sensor performance based on selectivity parameters. Testing on all pH levels will minimize the interfering effects and determine the optimal conditions for metal detection. Hence, specific colour responses resulting from an optimal condition will only indicate the presence of a specific metal. A masking mechanism and sample pre-treatments may also be required to eliminate interference and improve selectivity. Khaodee et al. reported that Al^3+^ and Cu^2+^ were found to interfere with the detection of Pb^2+^ and Fe^3+^ using cyanidin, and they studied the use of masking agents as the solution to the problem (Table 3). The agents were expected to interact with the interfering metal ions to form colourless complexes. Al^3+^ ions were found to be completely masked by potassium fluoride for the determination of Fe^3+^ and Pb^2+^ at pH 4 and 6, respectively. Furthermore, dimethylglyoxime (DMG) showed potential of masking Cu^2+^ ions by forming Cu-DMG complex for the determination of Al^3+^ at pH 5 [106]. Some references also reported simple pre-treatment steps during application to real samples. Filtration is mostly used to remove insoluble materials [59,110,117]. Continuous improvements on sensitivity and selectivity to reduce false positives/negatives are essential to produce reliable methods.

## 8. Conclusions

Natural pigments can be useful for metal detection using colorimetric methods due to their colour-changing properties and pigment–metal complexes. According to various studies, based on their sensitivity (marked by the lowest LOD values), and by considering portability, it can be concluded that betalains are best applied for copper (Cu^2+^) using a smartphone-assisted sensor; curcuminoids are best for lead (Pb^2+^) using curcumin nanofibers; and anthocyanin is best for mercury (Hg^2+^) using an anthocyanin hydrogel. In addition, both curcumin and anthocyanin are also applicable for Fe^3+^. Each developed method gives a new perspective in the use of natural pigments for metal detection, either with or without instrumental analysis. However, it is noteworthy that selectivity and sensitivity improvements are the main parameters with regard to method-detection developments.

The practical uses of a developed method must be proven by direct testing on real samples, to determine that certain matrices do not interfere with the performance. Most of the studies mentioned above applied the developed methods to aqueous buffer media and water samples. Regardless of how many distributed pollutants are present, testing on other environmental samples should not be limited on water. Further research can expand experiments on matrices and organisms that are prone to heavy metals accumulation accompanied by developing masking agents to improve sensor selectivity. On the other hand, for the development of portable and on-site detection methods, a coloured sheet representing different metal concentrations can be useful as a colour standard, not only helping to provide qualitative results for fabricated forms, but also for quantitative estimations for metal contaminants that are above the limits of detection of the method.

## Figures and Tables

**Table 1:** The effect of metal ions on betalain stability and its potential metal-detector properties.

Pigment	Sample		Metal Selectivity	Colour Change	Stability	Limit of Detection	References
	Real	Synthetic					
Red beet extract (*Beta vulgaris* f. *rubra* L.) (solution)	Not tested	Mixture of acetic acid 0.7% and NaCl 0.25%	Cr^3+^	Decreased colour intensity (red) ^1^	Changes in the colour intensity at 540 nm at pH 3.89	Not determined ^2^	[68]
Red beet juice and pure betanin (solution)	Not tested	Phthalate buffer 0.1 mol and NaOH 0.1 mol	Cu^2+^	Decreased pigment retention ^1^	2.5 mmol metal concentration and higher decreased betanin retention	Not determined ^2^	[69]
			Fe^3+^	Decreased pigment retention ^1^		Not determined ^2^	
Betanin isolate from red beet root (solution)	Not tested	Acetic and phosphoric buffer	Ni^2+^	Decreased pigment absorbance ^1^	0.06 mM Ni^2+^ addition at pH 7–8 causes significant λmax shift	Not determined ^2^	[29]
			Cu^2+^	Decreased pigment absorbance ^1^	0.04 mM Cu^2+^ at pH 4 causes significant λmax shift	Not determined ^2^	
2-decarboxy-betanin pigment (solution)	Not tested	Organic solvent (EtOH, MeOH, added buffer acetate (3–5.5) and phosphate (5.5–8)	Cu^2+^	Decreased pigment retention ^1^	650 µM Cu^2+^ causes significant changes in UV spectra	Not determined ^2^	[70]
Beet root extract, betacyanin gold (AuNP) and silver nanoparticles (AgNP) (solution)	Tap water	Hg(NO_3_)_2_ in water	Hg^2+^	Red to violet (AuNP), yellow to purple (AgNP)	Colour stability not tested	25 µM (by fluorescent sensing at 380 nm)	[71]
Red beet extract (solution)	Drinking water	CuCl_2_ in water	Cu^2+^	Purple to orange-red	Pigment solution stable at pH 9	0.84 µM (linear graph estimation)	[58]

^1^ Naked eye observation was not reported; ^2^ Stability study.

**Table 2:** Studies on metal ion detection by curcuminoids.

Pigment	Sample		Metal Selectivity	Colour Change	Stability	Limit of Detection	References
	Real	Synthetic					
Curcumin gold nanoparticle	Not tested	Hg^2+^ in phosphate buffer solution (pH 7.4)	Hg^2+^	Reddish wine to light blue	Nanoparticles are stable at pH 7.4 Colour stability not tested	2–10 µM (visual)	[86]
Curcumin-loaded zein membrane	Drinking water, tap water, pond water with pre-treatment of nitric acid	Fe^3+^ in deionized water with nitric acid (pH 2)	Fe^3+^	Yellow to brown	High efficiency for visual sensing at pH 2	7.16 µM (visual)	[59]
Curcumin-loaded cellulose acetate sensor strip	Not tested	Lead acetate solution at pH 5	Pb^2+^	Yellow to orange	Colour stability not tested	20 µM (visual), 0.12 ± 0.01 µM (linear graph)	[30]
Nanoparticle Ag + Curcumin (AgNPs-CUR) (solution)	Drinking water, aquarium water, tap water	Pb^2+^ solution in deionized water	Pb^2+^	Yellow to orange	Calculated binding energy of AgNP-CUR and Pb^2+^ is −361.4 kcal mol^−1^ (highly stable)	13.6 µM (linear graph estimation)	[90]
*Aloe barbadensis*, *Musa acuminata x balbisiana*, Curcumin biofilm	Water	Fe^2^ solution in Milli-Q water	Fe^2+^	Yellow to greenish brown	Colour stability not tested. Sensor biodegradability rate of 1.28% in one week	49 µM (linear graph estimation)	[88]
Curcumin nanoparticle immobilised starch cryogels	Tap water with pre-treatment of nitric acid	Fe^3+^ solution in ultrapure water at pH 2	Fe^3+^	Yellow to red brown	Colour stable at pH 2. Sensor is stable for 3 months in a desiccator	8.59 µM (linear graph estimation)	[92]
Curcumin bacterial cellulose nanofiber	Rice with pre-treatment of nitric acid, hydrogen peroxide, acetate buffer solution at pH 5	Lead acetate solution in Milli-Q water at pH 5	Pb^2+^	Orange to red	Yellow colour stable at pH 5. Sensor stability not reported	9 µM (visual), 0.9 µM (image processing)	[91]

**Table 3:** Studies on metal ion detection by anthocyanins.

Pigment	Sample		Metal Selectivity	Colour Change	Stability	Limit of Detection	References
	Real	Synthetic					
Cyanidin red cabbage extract (solution)	Pond water and tap water with pre-treatment of nitric acid and masking agents of potassium fluoride and dimethylglyoxime	Cu^2+^/Pb^2+^ nitrate-salt and Al^3+^/Fe^3+^ chloride-salt in Milli-Q water with buffer solution (pH 3–7) and masking agents	Cu^2+^	Violet to blue	Colour change at pH 7	50 µM (visual)	[106]
			Pb^2+^	Purple to violet to blue	Colour change at pH 6	80 µM (visual)	
			Al^3+^	Purple to violet to blue	Colour change at pH 5	50 µM (visual)	
			Fe^3+^	Pink to violet to blue	Colour change at pH 4	200 µM (visual)	
Anthocyanin extract from *Ruellia tuberosa* L. (solution)	Tap water and pond water with pre-treatment of nitric acid	Fe^2+^ solution in deionized water with nitric acid at pH 1	Fe^3+^	Pink to red	Anthocyanin extract stable at 4 °C for 2 months Red colour is stable at pH 1	0.54 µM (linear graph estimation)	[31]
Chitosan nanoparticles and cyanidin-based red cabbage extract (dipstick sensor)	Not tested	Fe^3+^ solution in distilled water with phosphate buffer solution at pH 4–6	Fe^3+^	White to pink	Cyanidin based nanoparticle highly selective at pH 4–6. Sensor stability not tested	179–7162 µM (visual)	[60]
Curcumin-anthocyanin hydrogel strips	River water with pre-treatment of filtration	Cd and Hg chloride salt in triple distilled water	Cd^2+^	White to bluish green	Not reported	0.2 µM (visual)	[110]
			Hg^2+^	White to blue	Not reported	0.2 µM (visual)	

## Data Availability

All data is contained within the article.
